# The Evolution of Illicit-Drug Detection: From Conventional Approaches to Cutting-Edge Immunosensors—A Comprehensive Review

**DOI:** 10.3390/bios14100477

**Published:** 2024-10-03

**Authors:** Nigar Anzar, Shariq Suleman, Yashda Singh, Supriya Kumari, Suhel Parvez, Roberto Pilloton, Jagriti Narang

**Affiliations:** 1 Department of Biotechnology, School of Chemical and Life Science, Jamia Hamdard University, New Delhi 110062, India; nigarsheikh111@gmail.com (N.A.); shariqsuleman07@gmail.com (S.S.); yashusingh2398@gmail.com (Y.S.); supriyapathakkumari@gmail.com (S.K.); 2 Department of Toxicology, School of Chemical and Life Science, Jamia Hamdard University, New Delhi 110062, India; sparvez@jamiahamdard.ac.in; 3 National Research Council, Department of Chemical Sciences and Materials Technology, Institute of Crystallography, 00015 Rome, Italy

**Keywords:** immunosensors, illicit drugs, conventional methods, point-of-care testing methods

## Abstract

The increasing use of illicit drugs has become a major global concern. Illicit drugs interact with the brain and the body altering an individual’s mood and behavior. As the substance-of-abuse (SOA) crisis continues to spread across the world, in order to reduce trafficking and unlawful activity, it is important to use point-of-care devices like biosensors. Currently, there are certain conventional detection methods, which include gas chromatography (GC), mass spectrometry (MS), surface ionization, surface-enhanced Raman spectroscopy (SERS), surface plasmon resonance (SPR), electrochemiluminescence (ECL), high-performance liquid chromatography (HPLC), etc., for the detection of abused drugs. These methods have the advantage of high accuracy and sensitivity but are generally laborious, expensive, and require trained operators, along with high sample requirements, and they are not suitable for on-site drug detection scenarios. As a result, there is an urgent need for point-of-care technologies for a variety of drugs that can replace conventional techniques, such as a biosensor, specifically an immunosensor. An immunosensor is an analytical device that integrates an antibody-based recognition element with a transducer to detect specific molecules (antigens). In an immunosensor, the highly selective antigen–antibody interaction is used to identify and quantify the target analyte. The binding event between the antibody and antigen is converted by the transducer into a measurable signal, such as electrical, optical, or electrochemical, which corresponds to the presence and concentration of the analyte in the sample. This paper provides a comprehensive overview of various illicit drugs, the conventional methods employed for their detection, and the advantages of immunosensors over conventional techniques. It highlights the critical need for on-site detection and explores emerging point-of-care testing methods. The paper also outlines future research goals in this field, emphasizing the potential of advanced technologies to enhance the accuracy, efficiency, and convenience of drug detection.

## 1. Introduction

Excessive usage of illicit drugs due to the euphoric stimulation, which over-activates the “reward circuit” of the brain, has terrible repercussions on people’s health, the economy, and communities around the world. Despite the ongoing “war on drugs”, it remains a significant problem [1]. It is challenging to estimate the rate of increase in the use of psychotropic chemicals illegally provided for non-medical purposes, but it appears to be expanding in many regions of the world. It is challenging to determine how often this occurs. It is difficult to gage the behavioral and negative effects on health that it has in certain populations as it is illegal and thus, frequently concealed [2]. Due to the stress and pressure of modern life, individuals are becoming increasingly susceptible to drug abuse, especially youngsters, who are more prone to the issue. Their addiction negatively impacts society’s mental, physical, and social well-being, sometimes resulting in serious crimes like sexual assaults, robbery, accidents, and even murders [3]. Cocaine, marijuana, gamma-hydroxybutyrate (GHB), methylamphetamine (MAMP), lysergic acid diethylamide (LSD), ketamine, amphetamine (AMP), and others are some of the illicit drugs that are abused. Some of these drugs mimic the brain’s chemicals, known as neurotransmitters, like serotonin, and send abnormal signals; while others release excessive natural neurotransmitters such as dopamine, which affects the signal communication between the neurons and can lead to hallucinations and paranoia. Ketamine, gamma-hydroxybutyrate (GHB), and Rohypnol are agents in drug-facilitated sexual assault (DFSA) [4]. In order to manage these drugs, the key is to detect them as early as possible. There are many methods of detection but they all lack one or more key features such as being less sensitive and specific, require expensive equipment, etc. To cope with this issue, there is a need for a point-of-care device such as a biosensor that can offer real-time results. A biosensor is an analytical device that incorporates a biological sensing element with a transducer to detect and measure a specific analyte. Biosensors can be designed with specific antibodies or aptamers that selectively bind to illicit drugs. When the drug binds to the sensor, it triggers a measurable signal, such as a color variation or electrical response, indicating the drug’s presence [5].

Immunosensors are distinguished by their remarkable sensitivity and specificity because they employ antibodies or antigens as the recognition component. Their ability to differentiate between closely similar molecules owing to their specificity lowers false positive rates and assures reliable findings. Their adaptability makes them useful in a variety of industries, including environmental monitoring and healthcare, by enabling the detection of a broad spectrum of analytes. In addition, immunosensors provide label-free detection alternatives, long-term stability, and real-time monitoring capabilities, all of which enhance their efficacy and dependability in highly accurate and efficient target chemical detection [6,7]. Immunosensors are defined as affinity-ligand-based biosensors and work on the principle of an immunochemical reaction coupled with the transducer to give a rapid and high-sensitivity response with convenience of usage and low cost. These can be used in the recognition of pathogens, toxins, antibodies, biomarkers, and illicit drugs. Much of the success of immunosensors can be attributed to the development of POCT techniques with different types of transducers specific to the sample and its intrinsic properties and quantity [8]. In recent times, mental health has become a major concern with the rapid rise in the cases of drug addictions. Immunosensors can also be used in the early evaluation of anxiety or depression even at a molecular level by using functionalized polyaniline carbon-based integrated quantum dots with sensitive electrochemical immunosensing by determining HSP70 (Heat Shock protein 70, which is considered as the depression marker) within a range of about 0.0976–100 ng/mL and LOD of 0.05 ng/mL [9]. Modification of chemicals, isomerization, prodrug formulations, encapsulation, and combination with other drugs are some strategies used by drug users/suppliers to conceal the presence of illegal drugs. These strategies make it more difficult for forensic laboratories and law enforcement to correctly identify these drugs [10]. The article outlines the commonly used illicit drugs, their effects on human health, and traditional/conventional methods used to detect illicit drugs along with their characteristics. Of greatest significance, it covers the latest developments in immunosensor types and detection methodologies.

## 2. Commonly Used Illicit Drugs and Their Adverse Effects on Human Health: The Risks Are Real

Illicit drugs are difficult to detect due to their designed chemical structures, which evade detection methods. Manufacturers constantly adapt formulations to outpace detection efforts. These tactics challenge attempts by law enforcement and forensic labs to identify these substances effectively. Long-term usage of drugs that are inhalants tends to injure or destroy nerve cells either in the brain or the peripheral nervous system, which can result in mental disorders, especially in people suffering from psychiatric disorders. SUDs (substance use disorders) create antisocial personality disorders including bipolar, psychotic, depression, and anxiety disorders [11]. The detailed structures of these drugs and their effect on human health are described below.

### 2.1. Methamphetamine

**Figure 1:**
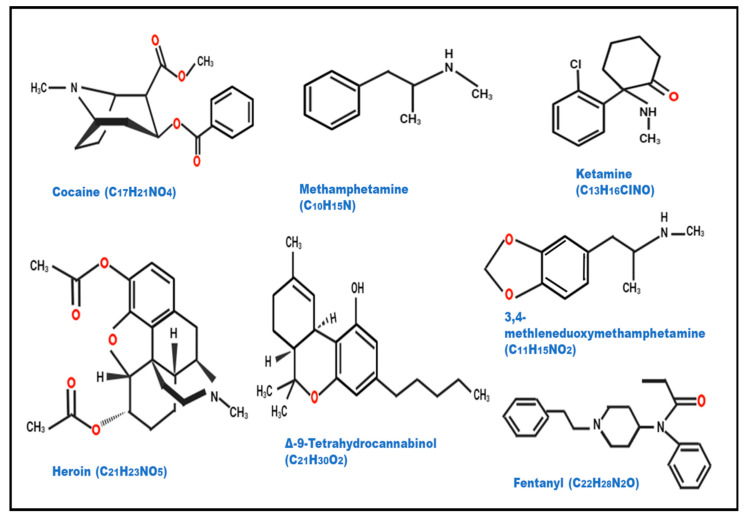
Chemical structures of some illicit drugs.

Methamphetamine (C_10_H_15_N) is a stimulant with a chemical structure consisting of a benzene ring fused to an amphetamine group (Figure 1). It is a member of the amphetamine class and a derivative of amphetamine. It functions as a sympathomimetic drug, which means that it interacts with receptors in the sympathetic nervous system to imitate endogenous signals. It is frequently produced illegally using a mixture of substances, including pseudoephedrine and ephedrine. It comes in tablet, crystalline, glass-like, rock-like, or powder form. Meth can be snorted, smoked, or injected depending on its form [12]. Methamphetamine usage for a brief period of time can cause a number of physical side effects, such as headaches, dizziness, dry mouth, nausea, and hyperthermia. However, long-term usage raises the possibility of psychosis or psychotic symptoms. Furthermore, long-term usage of methamphetamine is linked to certain physical consequences of inadequate sleep and diet, namely, weight loss, and respiratory illnesses. At the same time, methamphetamine is a legitimate therapeutic option for treating narcolepsy, obesity, attention deficit disorder (ADD), attention deficit hyperactivity disorder (ADHD), and overeating issues [13,14].

One study describes the binding of methamphetamine with MD-2, which is a co-receptor of TLR-4, contributing to the extracellular increase in dopamine levels [15]. Methamphetamine possesses a variety of physiological properties, which makes it challenging to determine its presence in the human body. The body extensively breaks down methamphetamine by procedures such as N-demethylation, aromatic hydroxylation, and deamination, which are primarily carried out in the liver. Several metabolites are generated as a result of these metabolic processes, some of which are pharmacologically active, making detection more difficult [16].

### 2.2. Cocaine

Cocaine is a white crystalline alkaloid compound procured from *Erythroxylum coca* (coca leaves), which is grown all over the globe, especially in Peru, Bolivia, and Ecuador. Its chemical structure (C_17_H_21_NO_4_) consists of a benzene ring (aromatic ring) fused to a 2-carbomethoxy-3-phenylpropane (tropane) moiety (Figure 1). Cocaine’s chemical structure contributes to its pharmacological effects, including its stimulant properties on the central nervous system. It is a naturally occurring potent stimulant drug that causes addiction and directly affects the central nervous system [17]. Users inhale cocaine powder or inject it in solution form into the bloodstream. Cocaine has a high binding affinity of about 90% to albumin and α1-acid glycoprotein. It predominantly distributes in the kidney, brain, brain, lungs, and spleen with lesser amounts in the blood, heart, and muscle tissues [18]. Cocaine has an average half-life of about 40–90 min in the human body but duration is also influenced by the route of administration, being typically shorter for intravenous use and longer for inhalation. After consumption, cytochrome enzymes quickly break down THC in the liver, producing the pharmacologically active 11-hydroxy-THC and the inactive 11-nor-9-carboxy-9-tetrahydrocannabinol metabolites. The synthesis of 11-OH-THC is catalyzed by CYP2C9, and it can oxidize further to become 11-oxo-THC before becoming THC-COOH. This procedure has a key role in the euphoric effects of oral cannabis use. Within 72 h, about 70% of THC is eliminated, mostly through urine and feces, with THC-COOH being the most common urinary metabolite. Urine contains traces of unaltered THC but only very little 11-OH-THC (less than 2% of the total dose). Cocaine addiction is the leading source of global public health and economic issues, with cocaine being the most widely used illegal drug in Europe and the United States after cannabis [19].

### 2.3. Heroine

Heroin (C_21_H_23_NO_5_) is a strong opioid that consists of morphine (obtained from poppy plant seed pods) with two acetyl groups joined to the morphine nucleus (Figure 1). Because of its structure, heroin interacts with opioid receptors in the brain and central nervous system to provide potent analgesic and euphoric effects. It is commonly found in white or black powder, the latter known as black tar in the market, and creates a rush inside the body and clouds mental functioning. It can be smoked, snorted, inhaled, or injected. Its effect starts rapidly and lasts for about 8–12 h. Short-term use of heroin can cause euphoria, pain relief, nausea, vomiting, and dry mouth [20]. Long-term use can lead to addiction, tolerance over time, infections, mental health issues, and many other effects. Heroin use can also result in hypoxia due to the slow breathing effect, resulting in coma or even permanent brain damage. Heroin is quickly metabolized into other substances, mainly 6-acetylmorphine (6-am) and morphine, both of which are detectable for short periods, typically up to 24 h in urine, after which only morphine is detectable. Due to their strong lipophilicity, heroin and its metabolites can quickly pass through biological membranes, such as the blood-–brain barrier. This characteristic makes it easier for the substance to spread quickly throughout different tissues and organs, which makes detection more difficult [21,22].

### 2.4. Cannabis

Cannabis, commonly known as Marijuana, is one of the most widely abused psychoactive drugs worldwide. Marijuana is an edible dried flower, and the leaves of the plant Cannabis sativa contain a chemical known as delta-9-tetrahydrocannabinol or THC (C_21_H_30_O_2_), which is the main psychoactive compound. Figure 1 shows the chemical structure of THC, which includes a pentyl side chain (C_5_H_11_) attached to a resorcinol moiety. THC interacts with cannabinoid receptors in the brain such as CB1 leading to a psychoactive effect. It has a huge impact on the mind by altering the thinking responses and is highly used in the market nowadays [23]. There are two main cannabis receptors found in the human body. Several cannabinoids, each with distinctive physiochemical characteristics, are present in cannabis. Cannabis products may also include additional cannabinoids, such as cannabinol (CBN) and cannabidiol (CBD), in addition to THC. The complexity of detection and quantification increases when many substances with different quantities and characteristics are present [24,25]. Smoking cannabis has severe effects on oral soft tissue, which results in 69.9% of users experiencing xerostomia, leucoedema, poor oral hygiene, periodontitis, and an increase in the density of candida albicans [26].

### 2.5. Fentanyl

Fentanyl (C_22_H_28_N_2_O) comes under the category of synthetic opioid illicit drugs and is considered 50 times more powerful than heroin or morphine, resulting in over 150 deaths per day due to its overuse according to a report published by the CDC. Fentanyl’s structure contributes to its strong analgesic properties but also poses a high risk of overdose due to its potency (Figure 1) [27]. Fentanyl is present in several forms such as powder, pills, and liquids, making it highly dangerous due to its potency and unpredictable concentrations. Strong synthetic opioids like fentanyl are utilized as narcotic analgesic supplements for both general and regional anesthesia, as well as for the treatment of severe, ongoing chronic pain. Fentanyl usually takes less than two hours to take action, and its onset is quick. The most typical fentanyl side effects are asthenia, disorientation, hallucinations, and sleepiness. The toxicity of Fentanyl and its analogs causes nervous system breakdown and other respiratory problems similar to those of morphine due to its structural similarity to morphine and higher empathy for μ-opioid receptors. Fentanyl is lipophilic, like many other opioids, which means it easily passes through biological membranes, such as the blood–brain barrier. This characteristic aids in its quick commencement of action, widespread diffusion throughout the body, and capacity to build up in adipose tissues, making it very difficult to detect [28].

### 2.6. MDMA

3,4-Methylenedioxymethamphetamine (C_11_H_15_NO_2_), popularly known as Molly, Mandy, or Ecstasy, acts as both a stimulant and a hallucinogen. MDMA’s structure contributes to its psychoactive effects by affecting serotonin, dopamine, and norepinephrine neurotransmitter systems in the brain. Ecstasy use for recreational purposes is a global issue (Figure 1). Ecstasy is commonly ingested orally as pills or capsules, while it can also be injected intravenously or inhaled. Because it is commonly used during dance events, especially “raves”, ecstasy is frequently referred to as a “club drug”. At the same time, some users use ecstasy during routine house parties, casual get-togethers with friends, etc. [29,30]. MDMA affects three main chemical messengers in the brain, namely, serotonin, norepinephrine, and dopamine, and the effects usually last up to 3 to 6 h, causing a rapid increase in energy, high body temperature, involuntary teeth tightening, and depression. Since MDMA dissolves in fats and oils, it is considered to be somewhat lipophilic. Because of this characteristic, MDMA may easily pass through cellular membranes, such as the blood–brain barrier, which causes its effects to manifest quickly and spread throughout the body [31,32].

### 2.7. Ketamine

Ketamine, frequently referred to by the name “special K”, is a general anesthetic that is utilized in human and veterinary procedures. It relieves pain, sedates, and causes memory loss in addition to inducing a trance-like condition. Ketamine can have hallucinogenic and dissociative effects when taken recreationally, which can result in feelings of being detached from reality or out-of-body experiences. It is categorized as a schedule III restricted drug in several nations, including the US, because of its potential for misuse. Ketamine acts on NMDA receptors, which are involved in mood and memory regulation, to alter brain function [11]. In comparison to other dissociative medications, its effects are comparatively fleeting; yet, excessive dosages may cause the user to enter what is colloquially referred to as a “K-hole”, a state of extreme dissociation and immobility. The chemical structure of ketamine, a synthetic substance, is characterized by a cyclohexanone ring linked to a phenyl ring. It is a member of the arylcyclohexylamine family of chemicals and has the molecular formula C_13_H_16_ClNO (Figure 1). Ketamine’s structure contributes to its pharmacological effects by blocking N-methyl-D-aspartate (NMDA) receptors in the brain, leading to its dissociative and anesthetic properties. Its pharmacological actions are facilitated by its distinct structure, which also affects metabolism and detection [33]. Ketamine is only detectable in urine for one to three days following usage due to its quick metabolism by the body. The detectable window in blood is much smaller, at about 24 h. It is broken down into a variety of metabolites during metabolism, such as norketamine, which can be somewhat long-lasting but is still difficult to track [34].

## 3. Conventional Techniques Available for Detecting Illicit Drugs

### 3.1. Gas Chromatography–Mass Spectrometry (GC-MS) and Liquid Chromatography–Mass Spectrometry (LC-MS)

GC-MS is a two-step analytical technique. The drug sample is first vaporized and then passed through a column in the gas chromatography (GC) phase. Here, various substances are sorted according to their chemical characteristics. Following their separation, the chemicals are sent into the mass spectrometry (MS) detector, where they fragment and ionize. The mass-to-charge ratios of these fragments are calculated, giving each material a distinct “fingerprint”. Similar to GC-MS, LC-MS uses liquid chromatography rather than gas chromatography for its separations. In this instance, materials are introduced into the mass spectrometer after being separated in a liquid solvent. For materials that cannot be vaporized in gas chromatography but are non-volatile or heat-sensitive, this method works perfectly. These techniques can easily distinguish between drugs with very similar structures, provide quantitative analysis, and are suitable for detecting drugs such as amphetamines, cocaine, opioids, and cannabinoids. Despite many advantages, their equipment and operational costs are very high compared to biosensors. Sample preparation and analysis is a quite lengthy process. They require lab facilities and trained operators [35,36,37].

Gas chromatography works under pressure while MS works under vacuum and can be used to analyze and detect the eluted unknown sample. GC-MS was used for the finding of illicit drug street samples in which THC and amphetamine were present in the highest quantity and, in almost 33% of the samples analyzed, caffeine was the adulterant that was most commonly present [38]. The method’s drawback is that the drug sample needs to be capable of being volatilized for separation and exploration. The other option is to analyze the sample using liquid chromatography–mass spectrometry (LC-MS), which is quite expensive and lacks universal commercial drug libraries if the sample cannot be put in the gaseous state. For these reasons, the majority of crime laboratories use GC-MS for the identification of illicit drugs [39]. In 2015, a study was published in which the presence of heroin and its metabolites was checked in umbilical cord tissue in humans due to an increase in the cases of Neonatal Abstinence Syndrome (NAS). This was determined using various techniques like ELISA and LC-MS/MS in which, out of 23,271 samples analyzed, 1773 specimens were shown to be morphine positive and 410 were also codeine positive [40].

### 3.2. Fourier Transform Infrared Spectroscopy (FTIR)

FTIR analyses determine the amount of infrared light a sample absorbs. Every substance has a different molecular structure that determines its infrared absorption pattern. The substance may be identified by comparing its absorption pattern, or spectrum, to a database. FTIR has the advantages that it can analyze solids, liquids, and gases, it can analyze samples quickly without destroying them, and it requires minimum sample volume. However, it has also disadvantages such as it does not show high sensitivity and requires a reference library [41]. The use of attenuated total reflection along with FTIR (ATR-FTIR), which increases sensitivity and selectivity, is the latest approach to detecting drugs. Using ATR-FTIR, 96 illegal drug seizures including methamphetamine (0.1–78.6%) were examined. A low limit of quantification of 0.3% for methamphetamine, a Root Mean Square Error of Prediction of 5.2, and an R^2^ of 0.9637 were shown [42]. To determine the chemical “fingerprint” of cocaine samples, a technique based on ATR-FTIR coupled with chemometrics was proposed [43]. Recently, 75% of the drugs in the “street sample” were quickly identified using FTIR, and the impact of cutting agents on identification (ID) was also examined. An evaluation of the MDMA detection limit revealed an accurate ID starting at 25% *w/v*. The capacity of FTIR to be used in concentration estimation was demonstrated by the correlation between concentration and the Hit Quality Index [44]. A validation study was carried out by a group of researchers, in which two supervised injection locations in Vancouver, Canada, used two point-of-care technologies: fentanyl immunoassay strips and Fourier Transform Infrared (FTIR) spectroscopy. The immunoassay strips demonstrated a sensitivity of 87.5% and specificity of 95.2%, with a false negative rate of 12.5% while, FTIR spectroscopy showed a sensitivity of 72.1% and specificity of 99.0%, with a false negative rate of 27.9% [45].

### 3.3. High-Performance Liquid Chromatography (HPLC)

In HPLC, drug molecules are separated using high pressure and a liquid solvent. A detector (such as a UV detector) is used to measure the concentration of the separated chemicals. HPLC provides quantitative analysis of drug concentrations and can detect a wide range of concentrations. Some of its drawbacks are that it is a time-consuming process, requires preparation and calibration, and equipment and reagents are costly. Moreover, HPLC and MS can be combined (LC-MS and GC-MS) for increased specificity [46]. In order to assess the drug components belonging to the cocaine group, opiates, amphetamine-like stimulants, and their metabolites in water samples, a polar endcapped reversed-phase (RP) column and direct injection (DI) of higher quantities were used in the development of an HPLC–MS/MS technique. Following validation that included investigations on matrix effect, recovery, sensitivity, linearity, and accuracy, the majority of pharmaceuticals were found to have limits of quantification (LOQ) of 0.2 ng L^−1^ in surface water and 20 ng L^−1^ in wastewater (WW) [47]. Using 33 deuterated standards, an analytical method was investigated for the simultaneous determination in human serum of 43 commonly used drugs of abuse and their metabolites from various chemical and toxicological classes, including amphetamines, benzodiazepines, dibenzazepines, cocaine, lysergic acid diethylamide, opioids, phencyclidine, tricyclic antidepressants, and zolpidem. High-performance liquid chromatography coupled with tandem mass spectrometry operating in positive ionization mode was used for all studies. Up to 550 μg/L, all analytes underwent calibration. For EDDP, the limit of detection was 0.6 ng/mL, and for flunitrazepam, it was 13.7 ng/mL [48]. Recently, cocaine was detected on banknotes using multiple calibration techniques coupled with GC-MS. Street cocaine is considered to be polar and quite soluble in water in nature and its quantification was achieved using HPLC and UV at 230 nm, returning responses corresponding to 1.58 to 14.7 μg per note, with an average of 6.96 μg per note [49].

### 3.4. Capillary Electrophoresis (CE)

CE uses an electric field to separate drugs in a narrow capillary tube according to their size and charge. It is helpful for examining intricate combinations and tiny samples. CE is often used in forensic toxicology and drug testing to detect and measure drugs of abuse in a variety of biological samples, including blood, urine, saliva, and hair. CE is capable of distinguishing between different types of amphetamines, including methamphetamine and its metabolites, which are often abused substances. CE can detect trace amounts of drugs even in complex matrices, provides rapid analysis, requires minimal sample volume, and can analyze a wide range of drugs and metabolites [50]. Although capillary electrophoresis (CE) has many benefits, it is not without restrictions. One significant disadvantage is that, in comparison to methods like mass spectrometry, it has a very low sample loading capacity, which may restrict its sensitivity for identifying very low drug concentrations. Furthermore, CE is vulnerable to changes since it depends on exact control over experimental parameters (such as capillary temperature and buffer composition) to guarantee repeatability. Neutral chemicals, which do not move in an electric field absent certain alterations or additions, can likewise be difficult to separate using this approach. Finally, without derivatization or augmentation, the detection techniques commonly employed in CE, such as UV detection, may have poorer sensitivity for certain analytes. Normorphine, morphine, 6-acetyl morphine (6-AM), and codeine were detected at an excitation wavelength of 245 nm with a cut-off emission filter of 320 nm. This allowed for a quick and easy analysis using native fluorescence and capillary electrophoresis (CE) without the need for any derivatization steps. Detection limits were in the 200 ng/mL range [51].

### 3.5. Colorimetric Test Kits (‘Spot’ Tests)

In colorimetric assays, a small quantity of a drug is combined with a reagent that changes color when the drug is present. Each drug interacts differently with different reagents to generate unique colors. Color tests are suitable for detection across a variety of drug classes since they can react with a specific functional group found in the chemical. These tests are a good option for presumptive identification since they are inexpensive, portable, easy to use, and provide fast findings without requiring sample preparation [52]. The most used color test for identifying unknown substances is the Marquis reagent, which is also the first to be used in test sequences. In a study, presumptive identification of synthetic cathinones was carried out, employing three aqueous reagent solutions: copper(II) nitrate, 2,9-dimethyl-1,10-phenanthroline (neocuproine), and sodium acetate [53].

## 4. Immunosensors for the Detection of Illicit Drugs

**Figure 2:**
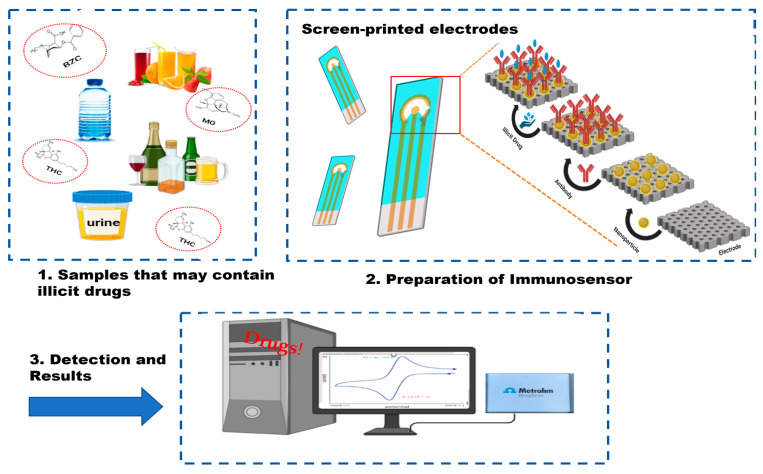
Representation of an immunosensor: illicit drugs in different spiked samples (1), screen-printed electrode for antibody-based detection (2), and electrochemical analysis using a potentiostat (3).

**Figure 3:**
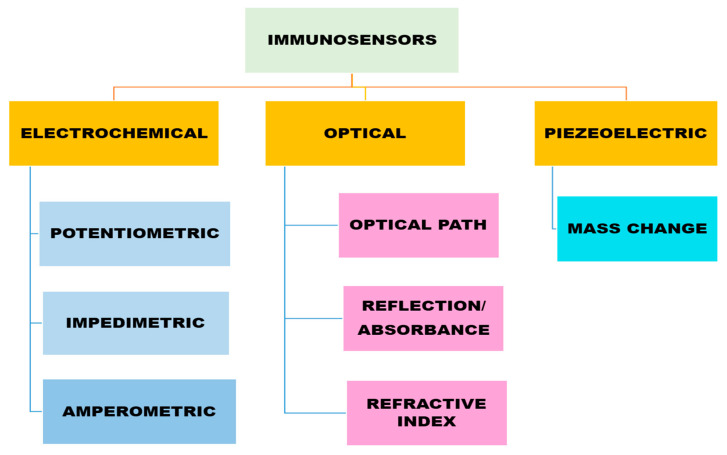
Classification of Immunosensors based on the transducer mechanism.

Conventional techniques possess several drawbacks such as the requirement for relatively large sample quantities and trained personnel, being expensive, bulky in size, and also having a time limit. But in real-life scenarios, we need devices that are user-friendly, i.e., can be used by someone who has not had specialized training, and that give fast and reliable real-time detection results. An immunosensor unit typically comprises a biological detection element, a signal transducer, and a signal monitoring system (Figure 2). In 1962, Leland C. Clark introduced the first enzyme electrode containing membrane-mounted glucose oxidase at the New York Academy of Sciences Symposium. This arrangement involves sensing molecules being coated or covalently attached to a probe surface. A membrane prevents interfering species from entering the analyte solution while retaining the detecting molecules. Sensing molecules detect substances and provide an electrical signal proportional to their concentration [54]. Immunosensors can be classified based on their transduction mechanisms, which convert the biological recognition of antigens and antibodies into measurable signals. Optical immunosensors detect changes in light characteristics by colorimetry, surface plasmon resonance (SPR), and fluorescence. Mass-based sensors pick up changes in mass on surfaces, whereas electrochemical immunosensors sense electrical changes like current or voltage. Figure 3 shows the different classifications of immunosensors along with their transducer mechanism. For a variety of applications in environmental monitoring, food safety, and medical diagnostics, each kind has unique benefits in terms of sensitivity and real-time tracking [55].

As explained by Gandhi et al. [56], based on the biological traits of opiates, different transducers are fabricated that work via signal transduction and property changes due to the formation of the Ag-Ab complex on the transducer surface. These transducers can be majorly classified as electrochemical (potentiometric, amperometry, conductometric, impedimetric), optical (luminescence, fluorescence, surface plasmon resonance—SPR), and piezoelectric immunosensors. Recognition elements can be natural (DNA, probe, aptamers, enzyme, tissue, microorganism) or artificial, including synthetic peptides and molecularly imprinted polymers (MIPs). Molecularly imprinted polymers (MIPs) are obtained by assembling a cross-linked polymer matrix around a template molecule that is held in a dispersive medium, either covalently or non-covalently, by judicious choice of functional monomer. Subsequent removal of the template molecular from the prepared polymer matrix produces a molecularly imprinted cavity with a shape matched to the template molecule [57]. The advantages and disadvantages of each type of immunosensor are summarized in Table 1.

Immunosensors give better sensitivity as shown in a study in which a novel fiber optic nano-plasmonic methamphetamine immunoosensor based upon competitive inhibition was developed. The sensor showed LOD of about 0.16 ng/mL with 15 min assay time. In another study, low concentrations of METH metabolites were detected from human urine or plasma samples with AgNP-based biosensors using colorimetric and fluorescence carbon nanodot detection techniques with LODs of about 0.82 μM and 0.1 μg/mL respectively, within 20 min [58]. Due to their sensitivity and selectivity, antibodies are frequently utilized in the construction of sensors for detecting illicit drugs. Employing highly specific antigen–antibody immunoreactions, immunosensors provide accurate analyte detection in complicated matrices. In this article, we will concentrate on the immunosensors that are used to identify illicit drugs. For example, trace amounts of cocaine were detected in water, urine, and body fluids by integrating electrochemical ELISA performed on anti-cocaine antibody with magnetic beads (Ab-MBs) into a microfluidic screen-printed electrode immunosensor developed by Abdelshafi et al. [59,60]. Researchers recently developed impedimetric immunosensors for the detection of on-site therapeutic drug monitoring of Rituximab (RTX) in blood serum and urine samples. Point-of-care (POC) biosensing systems have drawn interest due to their high specificity, portability, and, relatively low cost. They are used in the quick on-site analysis of illicit drugs [61]. An overall summary of immunosensors along with their limit of detection and assay time is presented in Table 2.

### 4.1. Electrochemical Immunosensors

Electrochemical immunosensors, which use an electrode system that changes the biological recognitional event into an electric signal, have the potential to become the core of POC technologies. Electrochemical immunosensor detection approaches can be classified into label-free (direct) and labeled (Indirect) assays, which can be the basis of competitive or non-competitive immunosensors. Generally, for large analyte sizes, direct immobilization of the antibody is carried out; however, a small analyte may induce structural changes, so in this case, the antibodies are conjugated with nanoparticles (Au-NPs) or different quantum dots (QDs) to avoid false results [62].

Researchers exploited the high binding affinity of MAM-functionalized hapten (MAM-COOH) to successfully develop a chemiluminescence immunoassay (CI) that is both extremely sensitive and dependable to identify heroin and its primary metabolites in urine samples. The concentrations of heroin, MAM, morphine, and codeine in spiked urine samples were found to vary from 0 to 1000 ng/mL, with detection limits of 80, 95, 90, and 75 pg/mL, respectively [63]. Furthermore, based upon the competitive enzyme-linked immunosorbent assay in conjunction with capillary arrays, researchers developed a quick, sensitive, and quantitative detection of drugs of abuse in sweat. It was demonstrated that four common drugs of abuse—methadone, methamphetamine, amphetamine, and tetrahydrocannabinol—could be detected in approximately 16 min with a wide dynamic range (methadone: 0.0016–1 ng mL^−1^; METH: 0.016–25 ng mL^−1^; amphetamine: 0.005–10 ng mL^−1^; THC: 0.02–1000 ng mL^−1^) [64]. By combining the benzoylecgonine (BE) antibody with the electroactive macromonomer (EDOT-BTDA-PPhe) containing poly-l-phenylalanine, researchers demonstrated a unique surface design. This device detected both BE and cocaine, acting as an electro-immunosensor platform for the investigation of cocaine consumption. An electrode made of glassy carbon was coated with EDOT-BTDA-PPhe in order to build the immunosensor. Following this, the polypeptide chains were used to immobilize the BE antibody. Electrochemical methods verified the surface alterations. The electrochemical detection of cocaine and BE as indicators of drug addiction was carried out using the immunosensor. Linearity was found for both analytes in the 0.5–25 μM range. Eventually, the suggested technique was effectively used to analyze artificial biological fluids [65]. A novel smartphone-based sensor has been created that can identify various illegal substances in samples of saliva. With great precision, this sensor can concurrently identify benzodiazepines (BZD), cocaine (COC), and amphetamines (AMP). Every sensor electrode is tailored to identify a particular drug; the limits of detection (LODs) for AMP, BZD, and COC are computed to be 4.3 ng/mL, 9.7 ng/mL, and 9.0 ng/mL, respectively. Because of its structural resemblance to amphetamine (AMP), methamphetamine (MET) exhibits limited cross-reactivity with the antibody surfaces compared to other interferences. The sensor was able to identify these compounds in genuine saliva samples from methamphetamine users as well as healthy individuals in clinical testing [66].

**Figure 4:**
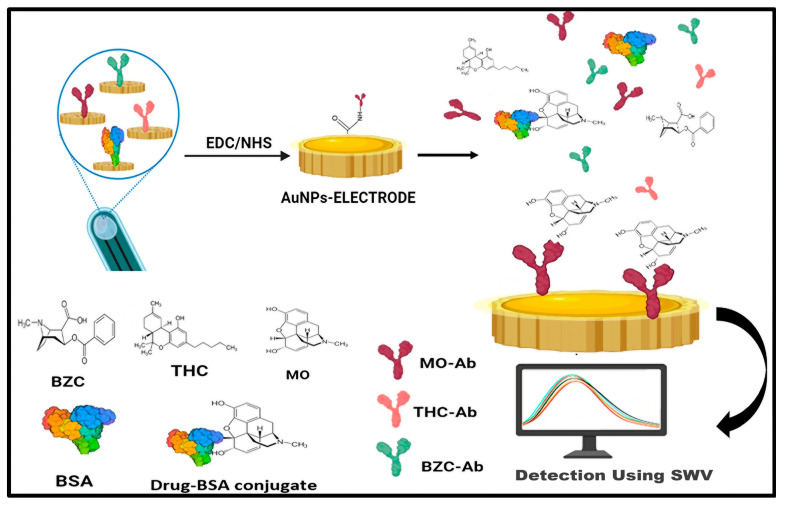
Multiplexed electrochemical immunosensor using a screen-printed electrode employing gold NPs for the detection of morphine (MOR), Tetrahydrocannabinol (THC), and benzoylecgonine (BZC) in spiked urine samples.

Screen-printed electrodes, which are simple, portable, low-cost, small in size, and can be mass-produced, employ a 30–40 µL sample volume for detection. These screen-printed electrodes can be used with multi-wall carbon nanotubes (MWCNT) produced by electropolymerization using cyclic voltammetry [67] and with molecularly imprinting polymer (MIP) modification for the rapid detection of Naloxone (NLX), which is a derivative of morphine and an opiate antagonist that can be used for the treatment of overdose [68]. A disposable screen-printed electrode was used for the detection of delta-9 tetrahydrocannabinol in undiluted saliva with the help of the electrochemical oxidation of Oxo245(N-(4-amino-3-methoxyphenyl)-methanesulfonamide), which showed a sensitivity of 28%, specificity of 99% and accuracy of 52% [69]. Another experiment was performed in which a multiplexed immunosensor consisting of eight electrodes was used for the analysis of urine to check for the presence of morphine (MOR), tetrahydrocannabinol (THC), and benzoylecgonine (BZC) within 20–40 min (Figure 4) by means of screen-printed carbon array electrodes altered with gold nanoparticles by square wave voltammetry. The concentration of MO and THC in the spiked urine samples was 10 ng/mL, while the measured levels using the compound-specific antibodies with PBS were found to be 11.51 ng/mL and 8.8 ng/mL, respectively, showing good sensitivity and selectivity. The detection limits were found to be 1.2 pg mL^−1^ for MOR, 7.0 pg mL^−1^ for 19-THC, and 8.0 pg mL^−1^ for BZC [70]. A rapid “swipe, scan, and detect” wearable electrochemical glove-based sensor for the detection of fentanyl in powder and liquid form, having a linear range of 10–100 μM, with a 10 μM LOD, was created by researchers [71] using square wave voltammetry (SWV), giving reliable on-site detection.

Hair is proving an impactful biological specimen for forensic toxicology analysis alongside urine or blood. To ensure effective biomarker detection while testing hair samples with immunosensors, specific extraction processes must be used. In contrast to other biological materials, hair needs to be extracted gently, avoiding the use of harsh organic solvents that can inactivate antibodies. Examples of these delicate extraction techniques include mechanical grinding or light chemical digestion. It is imperative that samples be prepared quickly, and techniques such as automated processes and optimized protocols can improve productivity. A disposable immunosensor based on electrochemiluminiscence was developed. The immunosensor’s viability was evaluated using spiked human hair samples. The hair samples were divided into sections after being cleaned. Hair segments and varying concentrations of the KET standard solution were broken down and neutralized in the presence of NaOH. The sensor showed a LOD of about 5.73 pg/g along with the advantages of low cost and a simple methodology [72,73].

In 1991, carbon nanotubes (CNTs) were first evidenced by Japanese electron microscopist Sumio Iijima (Iijima, 1991), and are now used as immunosensors due to their exceptional electrical, optical, chemical, and physical properties. They are extensively used for signal transduction for the recognition of analytes [74] and numerous illicit drugs. In 2018, a poly [3-(6-carboxyhexyl)thiophene-2,5-diyl]-functionalized carbon-nanotube-based chemiresistive sensor was fabricated by Zhang et al. for detecting meth in the air via its sensitivity to n-methylphenethyalamine (NMPEA), which is a detection stimulant of methamphetamine. The sensor can detect NMPEA concentrations as low as 4 ppb [75]. Recently, a single-walled carbon nanotube network was used for the trace-level analysis of fentanyl citrate with a detection limit of 11 nM withlinear range of 0.01–1 µM [76].

### 4.2. Optical Immunosensors

The new finer bundle optics called “Optodes” function as optical waveguides with advanced electronic capabilities, providing greater flexibility for use in medical applications [62]. Optical immunosensors are the most popular transducers for bio-analysis due to the advantages of employing visible radiation. Additionally, they work non-destructively and feature rapid signal generation output and reading. The two most important parameters for the development and application of immunosensors are high selectivity and sensitivity and optical biosensors play a great role in delivering these goals. A typical optical biosensor consists of a core often doped with germanium to increase its refractive index and a cladding, both made of silica, and works on the principle of TIR [77]. The optical immunosensor signal has low noise and is not generally disturbed by external disturbances, which is a great advantage over electrochemical-based immunosensors [78]. Optical immunosensors operate by detecting changes in optical properties such as absorption, fluorescence, and surface plasmon resonance phenomenon (SRP) at the surface of the sensor. These changes occur when an antigen-antibody complex forms. signal transducer due to the formation of an antigen–antibody complex forms [62].

In a recent study, a portable biosensor was reported. Exploiting the remarkable sensing capacity of G-protein coupled receptors, this yeast-based biosensor for real-life applications was constructed. The sensor was first used to screen a compound library to discover agonists and antagonists. It was then used to analyze 54 plants to discover a new phytocannabinoid, dugesialactone. Finally, it was developed into a robust portable device and used to analyze body-fluid samples and detect designer drugs like JWH-018. By using G-protein coupled receptors’ broad sensing repertoire, this technique may be expanded to detect a wide range of substances [79]. The electrons at the surface of the Noble metal nanoparticle react uniquely under incident light energy, which results in a change in collective oscillation as well as excitation level. This phenomenon, which acts at the interface between the dielectric material and metal, is known as surface plasmon resonance (SPR) [80]. The SPR of nanomaterials is tunable with changes in size, shape, and refractive index as well as an external environment so it can be used to make different types of biosensors [81]. Cannabis detection was demonstrated in paper-based sensing systems employing SPR. Through SPR, the immobilization of the antibody and its interactions with the target molecule complex containing 1–10 μg/mL THC were seen in an aqueous environment in situ. The results demonstrated the antibody’s strong attachment to the nanocellulose layer while maintaining its bioactivity [82]. A team of researchers developed a dual-channel, portable surface plasmon resonance (SPR) biosensor that can detect both MA and COC in saliva simultaneously and quickly. It is based on an indirect inhibitive immunoassay. On the sensor chip in each channel, antigens such as COC-BSA conjugates and MA–bovine serum albumin (MA-BSA) conjugates were produced and immobilized. The SPR signal was amplified by MA monoclonal antibody (MA-Ab) or COC monoclonal antibody (COC-Ab) upon interaction with the surface-immobilized antigens. The presence of MA or COC in the samples prevented the antibodies from attaching to antigens. Following optimization of the antigen and antibody concentrations, the resultant SPR inhibitive immunoassay demonstrated good sensitivity, with limits of detection for COC and MA of 3.14 ng/mL and 0.95 ng/mL, respectively [83].

**Figure 5:**
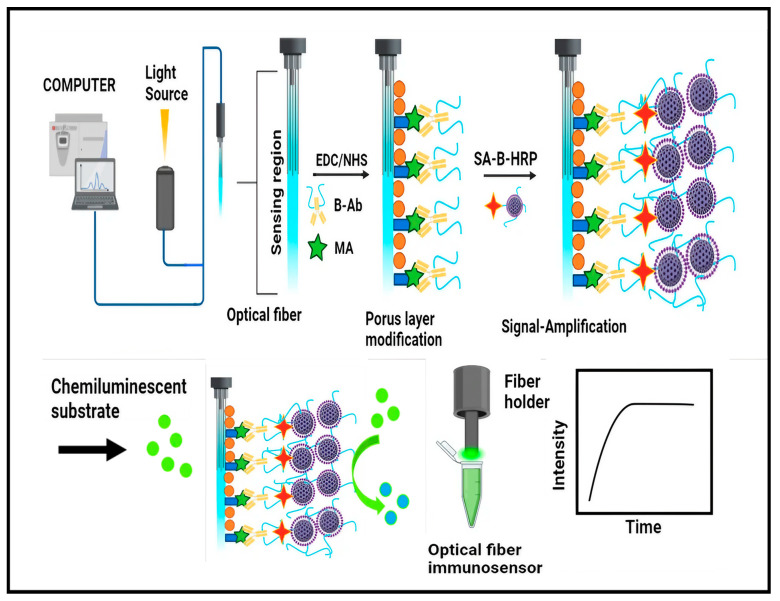
Schematic diagram for the fabrication of PCFS for MA detection in spiked biological fluids using SA-Bio-HRP nanocomposite.

A study was published that measures the change in fluorescence anisotropy i.e., when an antigen–antibody complex is formed there is a reduction in rotational freedom, which is retained. This information is now used to increase sensitivity and in the development of multichannel micro immunosensor chips without the problem of background noise of surface adsorption that can be used to detect illicit drugs [84]. With the advance of methods for drug marketing, modern biosensing progressively evolves from off-site laboratory tests to near-the-crime on-site diagnosis, which has recently led to the development of multiple point-of-care (xPOC), which combines several analyses on a single chip [85,86] via the integration of microfluidic channels with optical biosensors for the detection of illicit drugs [87,88]. The use of any illegal chemicals or techniques to improve athletic prowess, training, or performance is defined as “doping” by the World Anti-Doping Agency (WADA). For the identification and quantification of several compounds with various chemical and biological properties, doping tests are required. These optical fiber immunosensors perform excellently when evaluating substances for doping, namely, anabolic steroids, opioids, stimulants, and peptide hormones [89,90]. A portable chemiluminescent fiber-based immunosensor (PCFS) for sensitive quantitative and immune-specific identification of MA in biological sources was described by Zhau et al. For signal amplification, the PCFS integrates an optical fiber sensor with a biotin-streptavidin-mediated peroxidase nanocomposite and competitive enzyme-linked immunoassay. When combined with a portable battery-operated photon counting detector, a low detection limit of about 0.5 ng/mL was found. The PCFS is appropriate for quick and economical POCT of MA. Its linear range spans 1.5–300 ng/mL. With an analytical period of 10 min, the approach was effectively used in determining the amount of MA in a variety of biological materials, including human blood, urine, and oral fluid (Figure 5) [91].

### 4.3. Piezoelectric Immunosensors

Piezoelectricity refers to the phenomenon of a certain material, generally an anisotropic crystal, generating a voltage when they are mechanically stressed and vice-versa. When a piezoelectric material such as aluminum nitride, barium, crystallized topaz, quartz (SiO_2_), or berlinite is placed in an electric field, the crystal undergoes a process of deformation and starts to oscillate, which is further measured [92]. Piezoelectric biosensors are not only used in illicit drug detection but also in the identification of breast cancer biomarkers like alpha-fetoprotein (FPA) and prostate-specific antigen (PSA) as described by Ramirez-Vellas et al. [93]. The principle behind piezoelectric sensing is that piezoelectric materials like quartz, topaz, and barium oscillate in the presence of an alternating current; when the analyte binds to the antibody present in the piezoelectric material, the mass of the electrode increases, which results in a time-dependent frequency shift, giving a rapid, specific, highly-sensitive, and label-free detection system. The most widely known piezoelectric devices include Surface Acoustic Wave (SAW), Quartz Crystal Microbalance (QCM), and Bulk Acoustic Wave (BAW) devices. The SAW sensor detects changes in mass on its surface, which affects the speed of the acoustic wave and quickly alters the frequency. In contrast, Bulk Acoustic Wave (BAW) and Quartz Crystal Microbalance (QCM) devices use a quartz crystal water positioned between two metal electrodes. These setups connect to an external oscillator, allowing the quartz crystal to vibrate at its resonance frequency [94]. An ultrasensitive label-free immunosensor was made for the quantification of ketamine based on a variation in the resonance frequency of a quartz crystal microbalance (QCM), which converts the mass change on its surface into a frequency change as the output signal, which was optimized by placing a KT antibody on the QCM surface. The sensor had a detection limit of about 0.86 pg/mL (S/N = 3) and was linearly correlated with KT concentration in the range of 1 to 40 pg/mL. With recoveries ranging from 91.8% to 108%, the resulting immunosensor was used to detect different dilutions of KT in spiked human urine. No pretreatment was required [95]. An affordable and simple terahertz (THz) metamaterial immunosensor in conjunction with nanoscale Au film metamaterial (AuF MM) is being developed in an attempt to detect the presence of chloramphenicol residues in milk. For the purpose of qualitatively evaluating CAP residues in milk, the interaction between the THz waves and the refractive index response triggered by the complexes formed on the AuF MM surface as well as the gold particles themselves causes a change in spectral transmittance. Increased THz wave transmission reduces milk matrix interference, allowing for acceptable detection limits of as low as 5 pg/mL [96].

**Figure 6:**
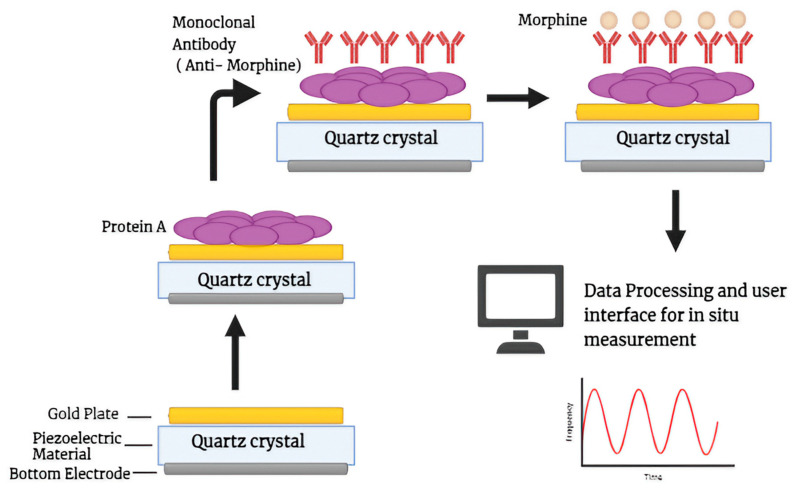
Schematic diagrammatic representation of piezoelectric immunosensor with quartz-gold nanoparticle altered electrode deposition for detecting morphine at different concentrations and temperatures.

Recently, a study published by Zahra et al. provides details about the fabrication of a morphine detector based on a 20 MHz piezoelectric quartz-Au biosensor using an anti-morphine monoclonal antibody operating at various temperatures, which can work in open and closed modes. This sensor can detect morphine with a limit of detection of 0.25 ng/mL, a limit of quantification of 0.25–2500 ng/mL, and a fast detection time of only 460 s (Figure 6) [97]. This immunosensor is also capable of regenerating with different morphine concentrations and can continue to detect for up to five regenerating steps. The great advantage of using these piezoelectric materials is the generation of piezoelectric-energy-harvesters commonly known as PEHs. These can be used in self-powered electrical applications in the field of medicine such as tissue regeneration and respiratory monitoring as well as nerve stimulation, which removes the disadvantages inherent in the use of lithium-ion-battery-powered devices, such as short life or discontinuity, an approach that is well explained in the study by Panda et al. [98].

**Table 1:** Comparative analysis of the feasibility of various immunosensing techniques in practical Applications.

Immunosensing Technique	Advantages	Disadvantages	Mitigation Strategies
Electrochemical	High sensitivity for detecting low concentrations of analytes. Rapid detection enabling real-time monitoring. Low cost and suitable for mass production [99].	Susceptible noise from external sources such as temperature, pH, etc. May require optimization of reaction times for antibody-antigen binding. Lower-cost material may affect stability or durability.	Careful preparation and use of redox mediator. Ensure accurate detection without compromising speed. Use of durable materials to improve longevity while maintaining low cost [100,101].
Optical	High sensitivity and selective, especially in fluorescence and chemiluminescence systems. Some optical methods (e.g., SPR, interferometry) allow label-free detection. Optical sensors can often detect both very low and high analyte concentrations.	Optical signals can be affected by ambient light or environmental conditions. Label-free detection may have lower sensitivity compared to labeled methods. Needs careful calibration to ensure accurate readings.	Use shielding or closed systems to minimize exposure to ambient light and control environmental factors. Use signal enhancement techniques, such as gold nanoparticles or nanostructured surfaces, to improve sensitivity in label-free systems. Implement calibration curves and software-based signal correction to extend the effective dynamic range [102,103].
Piezoelectric	Can detect mass changes directly without the need for labeling reagents. Allows real-time monitoring of antibody–antigen interactions. Can achieve high selectivity with specific antibodies or biomolecules.	Reduced sensitivity compared to optical or electrochemical sensors in detecting small analytes. Exact control of environmental factors may be required for dependable detection. Sensitivity might decrease in complex matrices.	Using signal amplification strategies such as nanomaterials to enhance mass changes. Use of temperature control and buffer optimization to stabilize environmental conditions. Use sample pre-treatment to eliminate interfering materials before detecting [104,105,106].

**Table 2:** Summary of different types of immunosensing techniques for detection of illicit drugs.

Drug	Technique Used	Sample Type	Limit of Detection (LOD)	Time	References
Cocaine	Multi-electrochemical competitive immunosensor using anti-cocaine antibody and protein-G-functionalized magnetic beads.	Urine, Serum, Saliva	14.4 ng/mL (urine) 3.6 ng/mL (Saliva) 25.2 ng/mL (Human serum) 3.6 ng/mL (saliva)	1–6 h	[107]
	Based on the combination of benzoylecgonine (BE) antibody and poly-l-phenylalanine-bearing electroactive macromonomer (EDOT-BTDA-PPhe)	Synthetic biological fluids	-	-	[65]
	surface plasmon resonance (SPR) biosensor based on an indirect inhibitive immunoassay	Saliva	MA and COC equal to 0.95 and 3.14 ng·mL^−1^	-	[83]
	Laser-induced immunofluorometric biosensor using high-affinity antibody IP3G2	Drug sample	0.023 ng/mL	90 s	[108]
	Microfluidic-chip-based electrochemical immunosensors integrated with ELISA	Oral Fluids	0.15 ng/mL	22 min	[60]
	Molecularly imprinted-polymer-based optical immunosensor using Mn-doped ZnS-QDs optosensing.	Oral fluids and serum	0.035 and 0.015 ng/mL for oral fluid and serum.	-	[109,110]
Morphine	Manufacture of morphine detector based on quartz@Au-layer piezoelectric immunosensor with mouse anti-morphine antibody.	Morphine sulphate	0.25 ng/mL	460 s	[97]
	Voltammetric immunosensor using screen-printed electrodes modified with gold nanoparticles	Saliva	0.09 ng/mL	-	[111]
	Label-free immunosensors based on ECL intensity of luminol using indium-tin coated glass with gold nanoparticle	Urine	0.82 ng mL^−1^	-	[112]
Fentanyl	An ultrasensitive monoclonal-antibody-based immunochromatographic strip along with ic-ELISA	Human urine and serum	0.14 ng/mL and 0.840 ng/mL	-	[113]
	Nanoporous electrochemical immunosensor for extremely sensitive detection employing gold working electrode and anti-fentanyl polyclonal antibody	sweat	11.5 ng/mL, after optimization can detect down to ≈1 ng/mL	-	[114]
Methamphetamine	portable chemiluminescent fiber-based immunosensor combined with optical fiber sensor with competitive enzyme-linked immunoassay employing biotin-streptavidin mediated peroxidase nanocomposite	biological sources (human blood, urine, and oral fluid)	0.5 ng/mL	10 min	[91]
	Electro-chemiluminescent immunosensor for POC testing using a portable meter.	METH-BSA	0.188 ng/mL	-	[115]
	Ionic liquid hydrogel material with increased sensitivity toward electrochemical detection	Saliva	0.72 ng/mL	-	[116]
	FOPPR technique based-biosensor for the detection using anti-MA bind to BSA-MA conjugate on gold NPs	Urine	0.16 ng/mL	15 min	[58]
Cannabis	Nanoimmunosensor based on Horseradish Peroxide and double-layer Gold Nanoparticles	Rat serum sample	0.0033 ng/mL	-	[117]
	Electrochemical Immunosensing employing surface of the GCE for anti-THC-monoclonal antibody	body fluids	0.0033 ng/mL	-	[118]
	A GPCR-based yeast biosensor	Artificial Saliva	-	-	[79]
Ketamine	A label-free immunosensor based on quartz crystal microbalance.	Human urine	0.0086 ng/mL	-	[95]
	Electrochemiluminescence immunosensor based on PAMAM.	Blood plasma	0.0067 ng/mL	-	[119]
	Electrochemiluminescence-based immunosensor using ketamine antibody immobilized on Au-NPs.	Human hair	0.0057 ng/mL	-	[73]

## 5. Other Detection Methods

Despite the tremendous advancements in immunosensor development, there are still significant obstacles to be tackled. In recent years, there have been reports of further point-of-care approaches. The development of sensing technology has led to the development of these techniques, such as MIPs. MIPs, or Molecularly Imprinted Polymers, serve as the recognition element that interacts with the target analyte, leading to measurable signals. MIPs can be used to create precise binding sites that match target molecules, which makes them useful for use in drug detection sensors. MIP-based sensors have great selectivity, sensitivity, durability, and cost-effectiveness, which makes them useful for quick on-site testing in public health monitoring and law enforcement. MIPs possess incomparable superiority relative to traditional analysis methods [120]. A low-cost potentially portable MIPs-based sensor was suggested that semi-quantitatively determines the presence of cocaine in oral fluid. A field collection device based on a cotton pad with an indicator and a molecularly imprinted polymer (MIP) sorbent was designed. Ion mobility spectrometry (IMS) was used to evaluate the extracted material, yielding a cut-off value of 20 µg L^−1^. The system was able to correctly identify 95% of true-negative and 100% of true-positive samples [121]. Aptamers have also gained a lot of attention recently due to their excellent selectivity and sensitivity, which make it possible to find illicit drugs in complex materials in low quantities. Aptamer-based sensors are useful for law enforcement and drug surveillance because they enable quick, on-site testing. Combining aptamers with nanomaterials in sensing and drug delivery in particular offers many advantages. This synergy enhances sensitivity by amplifying signals, allowing for the detection of lower target concentrations. Nanomaterials also increase the stability of aptamers, protecting them from degradation. Additionally, they improve selectivity, enabling better discrimination between target and non-target molecules. Furthermore, this combination allows for multiplexing, enabling the simultaneous detection of multiple targets. Researchers reported the simultaneous detection of METH and cocaine using a gold nanoparticle–conjugated aptamer sensor. High selectivity and sensitivity were obtained for both drugs in a single assay [122]. A simple cost-effective label-free biosensor was reported for detecting METH consisting of a G-quadruplex-hemin METH aptamer and colorimetric substrate. A low limit of detection of about 0.5 nm was achieved [123]. A colorimetric sensor was reported for detecting METH that used an aptamer as the recognition element and gold nanoparticles as the reporting probe, showing low LOD with good sensitivity [124]. A specific and rapid aptamer-based one-step method for detecting cocaine was also reported, in which aptamer bound the fluorescent molecule 2-amino-5,6,7-trimethyl-1,8-naphthyridine (ATMND) and thereby quenched its fluorescence. The sensor showed a low detection limit of about 200 nm [125]. The literature features a vast number of studies that made use of aptamers, MIPs, etc., as recognition elements and the results were satisfactory.

## 6. Conclusion and Future Aspects

Biosensors are fundamental tools that are used for the analytic recognition of Illicit drugs such as cocaine, marijuana, heroin, methamphetamine, etc. Over the years, several methods have been developed to reduce the menace created by the abundance of these psychoactive drugs and it is the biggest challenge of our century to create innovative methods to solve the upcoming social problems. Conventional techniques have major drawbacks as they are time-consuming, expensive, and require a lot of expertise to handle. However, immunosensors overcome these limitations through the mass production of cheap disposable sensors, real-time results, and multiple analyte analyses. In this review, different illicit drugs and their ill effects are detailed along with the conventional techniques used for their detection. The limitations of these conventional techniques are also detailed. Conversely, immunosensors are shown to have excellent features compared with conventional techniques and can detect compounds even in the nanometer range. However, at the same time, the most important challenge for immunosensors in detecting illicit drugs is selectivity. Achieving high specificity to the target drug without cross-reactivity to similar compounds is crucial, as false positives or negatives can compromise the accuracy of results, especially in complex biological samples like blood or urine. To tackle this issue, a lot of advancement is still needed in the future. This review emphasizes the recent advances in the development of different types of immunosensors involving single and multiplexed electrochemical immunosensors such as microfluidic, optical immunosensors using FOPPR (Fiber optic particle plasmon resonance), and quartz-based piezoelectric immunosensors using various fabrication mechanisms. It also describes point-of-care techniques for the rapid detection of substances of abuse in biological as well as spiked samples. The development of immunosensors using hydrogel, carbon dots, MIPs, or aptamers creates a great opportunity because of their high sensitivity, selectivity, and stronger binding affinity. In the future, the main challenge will be to meet the growing demand for more accurate and efficient point-of-care (POC) devices. At the same time, advancements in technology are expected to lead to the development of miniaturized, lower-cost, and faster devices for testing multiple illicit drugs simultaneously.
